# Systemic L-Kynurenine sulfate administration disrupts object recognition memory, alters open field behavior and decreases c-Fos immunopositivity in C57Bl/6 mice

**DOI:** 10.3389/fnbeh.2015.00157

**Published:** 2015-06-16

**Authors:** Dániel Varga, Judit Herédi, Zita Kánvási, Marian Ruszka, Zsolt Kis, Etsuro Ono, Naoki Iwamori, Tokuko Iwamori, Hiroki Takakuwa, László Vécsei, József Toldi, Levente Gellért

**Affiliations:** ^1^Department of Physiology, Anatomy and Neuroscience, University of SzegedSzeged, Hungary; ^2^Department of Neurology, Faculty of Medicine, MTA-SZTE Neuroscience Research Group, University of SzegedSzeged, Hungary; ^3^Department of Biomedicine, Graduate School of Medical Sciences, Kyushu UniversityFukuoka, Japan; ^4^Center of Biomedical Research, Research Center for Human Disease Modeling, Department of Physiological Sciences, Graduate School of Medical Sciences, Kyushu UniversityFukuoka, Japan; ^5^Faculty of Life Sciences, Kyoto Sangyo University, Kamigamo-MotoyamaKita, Kyoto, Japan; ^6^Department of Neurology, University of Szeged, HungarySzeged, Hungary

**Keywords:** L-Kynurenine, c-Fos, C57Bl/6 mice, open field, anxiety, novel object recognition, hippocampus, striatum

## Abstract

L-Kynurenine (L-KYN) is a central metabolite of tryptophan degradation through the kynurenine pathway (KP). The systemic administration of L-KYN sulfate (L-KYNs) leads to a rapid elevation of the neuroactive KP metabolite kynurenic acid (KYNA). An elevated level of KYNA may have multiple effects on the synaptic transmission, resulting in complex behavioral changes, such as hypoactivity or spatial working memory deficits. These results emerged from studies that focused on rats, after low-dose L-KYNs treatment. However, in several studies neuroprotection was achieved through the administration of high-dose L-KYNs. In the present study, our aim was to investigate whether the systemic administration of a high dose of L-KYNs (300 mg/bwkg; i.p.) would produce alterations in behavioral tasks (open field or object recognition) in C57Bl/6j mice. To evaluate the changes in neuronal activity after L-KYNs treatment, in a separate group of animals we estimated c-Fos expression levels in the corresponding subcortical brain areas. The L-KYNs treatment did not affect the general ambulatory activity of C57Bl/6j mice, whereas it altered their moving patterns, elevating the movement velocity and resting time. Additionally, it seemed to increase anxiety-like behavior, as peripheral zone preference of the open field arena emerged and the rearing activity was attenuated. The treatment also completely abolished the formation of object recognition memory and resulted in decreases in the number of c-Fos-immunopositive-cells in the dorsal part of the striatum and in the CA1 pyramidal cell layer of the hippocampus. We conclude that a single exposure to L-KYNs leads to behavioral disturbances, which might be related to the altered basal c-Fos protein expression in C57Bl/6j mice.

## Introduction

In the mammalian brain, more than 95% of the tryptophan is metabolized through the kynurenine pathway (KP) (Leklem, 1971). L-Kynurenine (L-KYN) is the central intermediate in this complex metabolic cascade, which ends with nicotinamide adenine dinucleotide, kynurenic acid (KYNA) and xanthurenic acid (Beadle et al., 1947; Heidelberg et al., 1949; Fujigaki et al., 1998). Certain kynurenines (e.g., KYNA) have been demonstrated to have neuroactive properties (Lapin, 1978; Perkins and Stone, 1982; Stone and Darlington, 2007; Vécsei et al., 2013). The *de novo* formation of KYNA from its precursor L-KYN is associated with the action of the kynurenine aminotransferases (KATs), and especially KAT II, which is located predominantly in the glial cells, but can also be found in the neurons (Guidetti et al., 1997; Rzeski et al., 2005; Lim et al., 2007). It is known mainly from *in vitro* studies that KYNA acts as a non-competitive antagonist on the α7 nicotinic acetylcholine (α7nACh) receptor at submicromolar level (Hilmas et al., 2001; Albuquerque and Schwarcz, 2013). It exerts dual action on the α-amino-3-hydroxy-5-methyl-4-isoxazole propionic acid (AMPA) receptor via two distinct mechanisms (Prescott et al., 2006; Rózsa et al., 2008), and at low micromolar concentrations (IC_50_ 15–240 μM, glycine concentration-dependent Hilmas et al., 2001) it can competitively antagonize the N-methyl-D-aspartate (NMDA) receptor, at the strychnine-insensitive glycine-binding site (Gál and Sherman, 1980; Birch et al., 1988). It has recently been demonstrated that KYNA is an endogenous ligand of the G-protein coupled receptor 35 (GPR35; EC_50_ 10 μM), which is expressed predominantly in the peripheral organs, but can also be found in the central nervous system (Wang et al., 2006; Cosi et al., 2011). KYNA can potently attenuate the amplitude of evoked excitatory post-synaptic currents at the CA1 pyramidal neurons in the hippocampus, via activation of the astrocytic GPR35 receptors (Berlinguer-Palmini et al., 2013). Moreover, GPR35 receptors can also be expressed by CA1 pyramidal neurons (Alkondon et al., 2015).

A shift in the brain concentration of KYNA has been described in several neurodegenerative disorders (Vamos et al., 2009; Schwarcz et al., 2012; Campbell et al., 2014). It decreases during epilepsy (Kamiński et al., 2003), Parkinson's disease (Oqawa et al., 1992; Szabó et al., 2011), and Huntington's disease (Beal et al., 1992, 1990), whereas it increases during schizophrenia (Schwarcz et al., 2001; Nilsson et al., 2005; Linderholm et al., 2012) and Alzheimer's disease (Baran et al., 1999; Gong et al., 2011).

The therapeutic application of kynurenergic manipulation was therefore proposed recently (Németh et al., 2004; Gigler et al., 2007; Stone and Darlington, 2007; Wonodi and Schwarcz, 2010; Gellért et al., 2011; Schwarcz et al., 2012; Tan et al., 2012; Stone et al., 2013).

Under physiological conditions, a systemic administration of L-KYN sulfate (L-KYNs) may result in the increment of several downstream metabolites of the KP [for instance the increased concentrations of quinolinic acid (QUIN) and 3-hydroxykynurenine (3-HK), neurotoxic components of the KP]. However, the most prominent change occurs in the concentration of the extracellular brain KYNA level, which dose-dependently increases in the striatum (Swartz et al., 1990), the prefrontal cortex (Zmarowski et al., 2009; Alexander et al., 2012) and the hippocampus (Scharfman et al., 2000; Wu et al., 2000), peaking at around 2 h following the injection. Concomitant region-specific decreases can be observed in the concentration of extracellular glutamate (Carpenedo et al., 2001; Alexander et al., 2012), dopamine (Rassoulpour et al., 2005; Wu et al., 2007; Olsson et al., 2012), acetylcholine (Zmarowski et al., 2009; Koshy Cherian et al., 2014), and gamma-aminobutyric acid (GABA, Beggiato et al., 2014).

In several studies done by our group and our contributors, L-KYNs administration proved to be neuroprotective in experimental models of neurodegeneration (Gigler et al., 2007; Knyihár-Csillik et al., 2007a; Robotka et al., 2008; Sas et al., 2008). In these studies, neuroprotection was achieved partly by the administration of 300 mg/bwkg L-KYNs. We have chosen the same dosage to obtain information about the effect of L-KYNs beyond neuroprotection in intact animals and to investigate how effects of this treatment converge on the level of behavior and c-Fos protein expression in mice. There is an unequivocal relationship between hippocampal c-Fos expression and memory formation (Vanelzakker et al., 2011). The relationship between basal ganglia activity and c-Fos expression is also described (Freeze et al., 2013). For this reason we targeted the hippocampus, which definitely corresponds to memory formation (Battaglia et al., 2011), and the striatum, which regulates movement velocity (Yin, 2014). We proposed that if altered behavior were observed, we would therefore find altered c-Fos expression as well.

There is available data on how acute kynurenergic manipulation alters behavior in adult rats. The long-lasting effect of pre-or perinatal kynurenergic manipulation in the rat is also partly described. Implementing similar experiments in mice is of particular importance, because such data is almost absent from the literature.

The ambulatory activity and anxiety-like behavior were assessed in an open field (OF) paradigm. Episodic-like memory performance was tested in an object recognition (OR) paradigm. The numbers of c-Fos^+^ cells were then compared in the corresponding brain areas by means of immunohistochemical technique.

## Materials and methods

### Animals

For the tests, 8–10 week-old male C57Bl/6j mice (*n* = 59) weighing 20–26 g were used. The animals were obtained from The National Institute of Oncology (Budapest, Hungary) and were housed under controlled laboratory conditions, in groups of 5, under an inverse 12-h dark/light cycle, with *ad libitum* access to food and tap water. To avoid the effects of shipping stress a 2-week habituation period was used before initiation of the behavioral testing (Walf and Frye, 2007). All housing and experiments were conducted in accordance with the European Communities Council Directives (86/609/ECC) and the Hungarian Act for the Protection of Animals in Research (XXVIII.tv. 32.§). Efforts were made to minimize the number of animals used and to reduce pain and discomfort. All of the experiments were approved by the following ethical license: XX/01593/I/2010.

### Drug administration

The mice were divided into two groups: the L-KYNs-treated animals (*n* = 30) received 300 mg/bwkg L-KYNs [dissolved in 5% NaOH and 0.2 M phosphate buffer (PB), pH 7.4] i.p., administered 2 h prior to the behavioral tasks or 3 h before the histological experiments, while the control animals (*n* = 29) were treated with the vehicle (0.2 M PB). All chemicals were purchased from Sigma, St. Louis, MO, USA.

### Locomotion activity measurement in an open field

The OF consisted of a square arena (50 × 50 cm) enclosed by continuous, 50-cm-high, light-gray opaque walls made of plexiglass. The apparatus was placed in a room illuminated by adjustable lamps giving a dim light within the arena (around 280 lux).

Mice (*n* = 9 per control group, 10 per L-KYNs-treated group) were placed into the middle of one side of the arena facing the wall. The animals were allowed to move freely for 8 min, while their horizontal ambulatory activity was tracked with the aid of a video-tracking system (SMART® by Panlab Harvard Apparatus). This allowed us to measure all the required parameters: total distance moved (cm), time spent moving (s), average speed (cm/s), number of entries into different zones and proportion of total time spent in the OF arena in different speed threshold ranges (percentage). Speed thresholds were correlated to the maximal moving speed (45 cm/s) of the mice, previously determined with an independent cohort of animals (previous study, not presented here). The maximal moving speed of the mice was then halved, to give a slow moving speed group (<22.5 cm/s) and a high moving speed group (>22.5 cm/s).

Following the experimental session, the mice were carefully removed from the OF, and returned to their home cage. The test equipment was cleaned with 50% ethanol solution and dried between subjects in order to avoid olfactory cuing.

### Behavioral observations in an open field

The anxiety-like behavior in the OF arena was assessed. The percentage of the time spent within the central part of the arena was determined, which was illuminated slightly better (300 lux) than the peripheral parts (250 lux). The central area delineated virtually with SMART® software, was taken as an imaginary inner square (30 × 30 cm) of the OF. The 8-min free exploration period was recorded simultaneously by a video recorder. Stereotyped behavior relevant at the level of anxiety (number of rearings, and times spent grooming and freezing) were scored manually (Carola et al., 2002). A single primary observer blind to the experimental condition conducted the behavioral observations.

### Object recognition

The OR memory task was performed in the OF arena, located in a testing room dimly lit by a constant illumination of about 50 lux in the test arena. The OF apparatus and the objects were cleaned with 50% ethanol solution and dried between subjects to avoid olfactory cuing. Unique objects were constructed from Lego® blocks that differed in shape and color (Supplementary Figure 1). These were around 10 cm high, and attached to the floor with Blu-Tack to avoid displacement by the animals. Duplicate copies of each object were used and each pair of objects was previously tested in the corresponding species for the absence of spontaneous preference for one object of the pair (unpublished observations). Within each experimental group, the role (familiar versus novel object) and the relative position of the two objects were counterbalanced and randomly permuted. Animals were placed in the experimental room at least 30 min prior to testing.

All animals (*n* = 10 per group) took part in a habituation session, when they could freely explore the OF for 5 min. No objects were placed in the box during this session. Twenty-four h after habituation, training was conducted by placing individual mice into the arena for 4 min, in which two identical objects (A and A1) were positioned in opposite corners, 7 cm from the walls. The amount of time spent exploring both objects A and A1 was recorded. The test session was performed 2 h after training, when the mice explored the OF for 4 min in the presence of one familiar (A) and one novel (B) object, and the time spent exploring the objects was recorded. Exploration of the objects was timed by a stopwatch when the mice sniffed, whisked or looked at the objects from no more than 1 cm away.

In order to analyze the OR performance of the mice, a modified version of a previously described formula (discrimination ratio Winters et al., 2008) was calculated as follows: novel × 100/(novel + familiar), where “novel” is the time spent exploring B and “familiar” is the time spent exploring A. This ratio named the discrimination index (DI), and shows the object exploration preference, expressed in percentage. Fifty percent denotes equal object preference, while higher values denote a preference for B, and lower values denote a preference for A.

### Tissue preparation

Animals were anesthetized (*n* = 10 per goup) with an overdose of urethane and perfused transcardially with ice-cold 0.1 M phosphate buffer (PB pH 7.4) and 4% paraformaldehyde (dissolved in 0.1 M PB, pH 7.4). The brains were removed and post-fixed overnight in 4% paraformaldehyde. On the next day, 20-μm coronal sections were obtained with a vibratome (Leica VT1000S) +0.54 mm and –2 mm from the bregma (MacKenzie-Graham et al., 2004). Five slices were collected in 100-μm steps from both regions.

### c-Fos fluorescent immunohistochemistry

In order to study possible alterations in neural activity caused by the elevated brain KYNA level, we used an indirect immunohistochemical method. 20-μm-thick free-floating sections were washed in PB, and then incubated in 1% normal donkey serum (NDS). For the detection of c-Fos-positive neurons in the striatum and in the hippocampus, sections were exposed to the primary antibody (rabbit anti c-Fos, 1:2000; Santa Cruz) overnight at 4°C, and for 2 h to the secondary antibody (Cy3 conjugated donkey anti-rabbit, 1:500; Jackson ImmunoResearch) at room temperature. Primary and secondary antibodies were diluted in 0.1 M PB containing 0.4% Triton-X100 and 1% NDS. The sections were coverslipped with antifade mounting medium (ProLong® Gold, Life Technologies). Fluorescent photomicrographs were obtained with an Olympus BX51 microscope fitted with a DP70 digital imaging system.

Changes in c-Fos protein expression occur within 30 and 90 min after certain forms of neuromodulation. A 3-h latency period was therefore interposed after vehicle or L-KYNs administrations, for the histological study.

### Quantification, data collection, and statistical analysis

In the CA1 region of the hippocampus, photomicrographs were captured in a frame of 500 × 140 μm at 200× magnification (**Figures 5A,B**). The dorsal part of the striatum included for analysis was captured at 100× magnification and delineated manually (Figures **6A,B**). c-Fos^+^ cells were automatically counted with custom-written software in MATLAB 7.1 (Mathworks, Natick, Massachusetts, USA). After automated threshold adjustment and noise reduction, fluorescent objects in the range 25–400 μm^2^ were accepted as cells and counted in binary images.

Numbers of c-Fos^+^ cells were compared with the Generalized Linear Mixed Model (GLMM). The data were regarded as overdispersed count data and negative binomial distribution with a log link was applied in the statistical analysis. The effects of the different mice were used as random effects and the different treatments were used as fixed effects in the mixed linearized model. Statistical analysis of the OF behavior was performed with the multivariate ANOVA (mANOVA). Robust Pillai's Trace multivariate test and bootstrapping analysis were used to handle violation of model assumptions if sample size was unbalanced. Statistical analysis of count data (number of rearings, see **Figure 3B**) was performed with Mann-Whitney *U*-test. Various statistical analyses were performed for evaluation of the OR performance. The paired *t*-test was used to compare the sample phase with the choice phase within each group, and the independent *t*-test was used to compare the same phases between groups (for the DI, see Section Object Recognition). The normality of the data was tested with the Shapiro-Wilk normality test.

All figures and computations were carried out with IBM SPSS Statistics software (version 20). Behavioral data were collected automatically with the SMART video-tracking system (PanLAB).

## Results

### Locomotion activity

**Figure 1:**
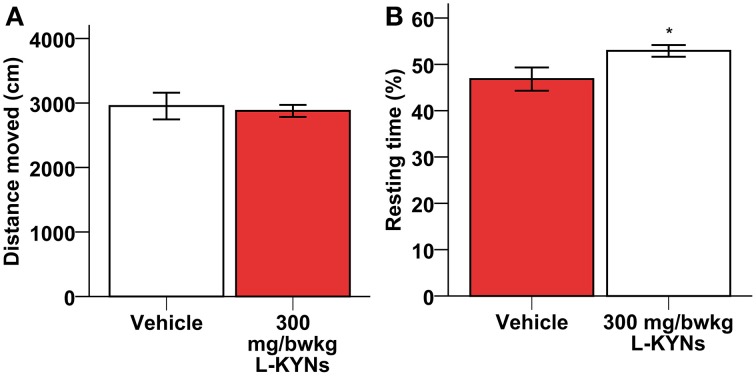
**Effects of L-KYNs treatment on the ambulatory activity of C57Bl/6j mice. (A)** Total distance moved (cm). No difference was found between the control and treated groups. **(B)** Percentage of resting time: treatment elevated the percentage of resting time. Data are shown as means ± SEM (mANOVA; ^*^p ≤ 0.05; *n* = 19 animals).

**Figure 2:**
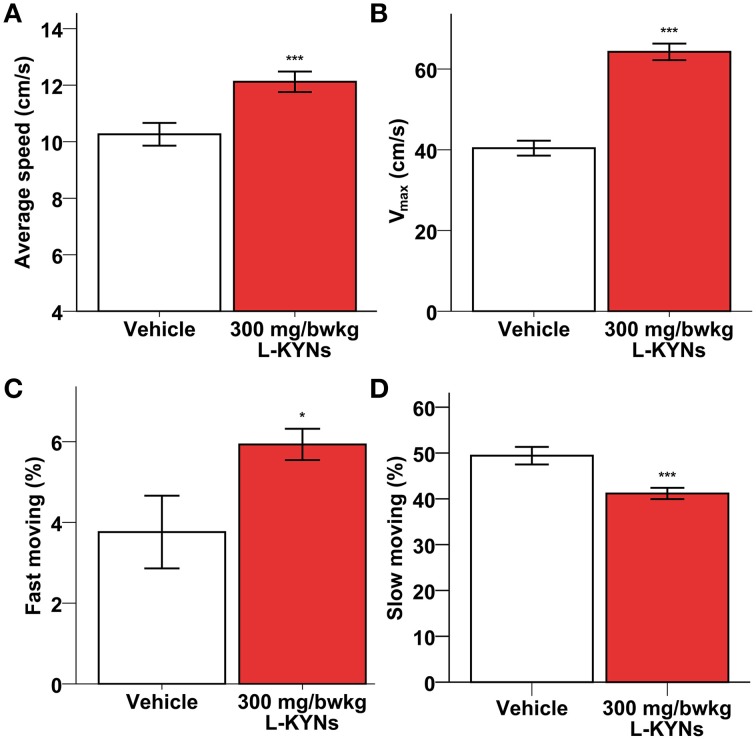
**Effects of L-KYNs treatment on the movement velocity of C57Bl/6j mice. (A)** Average speed (cm/s). Treatment significantly accelerated the average speed of the animals. **(B)** Maximal speed (cm/s). Treatment significantly elevated the maximal speed of the animals. **(C)** Proportion of high-speed movement (>22.5 cm/s). Treatment significantly elevated the percentage of fast moving. **(D)** Proportion of low-speed movement (<22.5 cm/s). Treatment significantly diminished the percentage of slow moving. Data are shown as means ± SEM (mANOVA; ^*^p ≤ 0.05; ^***^p ≤ 0.001; *n* = 19 animals).

A One-Way MANOVA revealed a significant multivariate main effect for treatment, Pillai's Trace = 0.977, *F*_(6, 11)_ = 78.766, *p* < 0.001, partial eta squared = 0.977. No difference in total distance moved was found between the control and L-KYNs-treated groups (Figure 1A; *p* = 0.91). However, the L-KYNs-treated mice moved for a significantly shorter time and their resting time was significantly elevated (Figure 1B; *p* = 0.032). Furthermore, the administration of L-KYNs significantly elevated the movement velocity of the C57Bl/6j mice as compared with the vehicle-treated group. Activation was observed in various parameters: their average speed (Figure 2A; *p* < 0.001), their maximal speed (Figure 2B; *p* < 0.001) and the proportion of high-speed movement (above 22.5 cm/s; Figure 2C; *p* = 0.020). Accordingly, the proportion of low-speed movement (under 22.5 cm/s) was significantly attenuated (Figure 2D; *p* < 0.001).

### Moving patterns

Although not confirmed by statistical data, the treatment altered the moving pattern of the animals. Their movement became wobbling and biphasic. In the “active” state, the mice accelerated and changed their direction rapidly and frequently. In the “passive” state, they stopped moving and expressed stereotyped freezing. These two states seemed to alternate randomly throughout the observations (Supplementary Video 1).

### Observations in the open field arena

**Figure 3:**
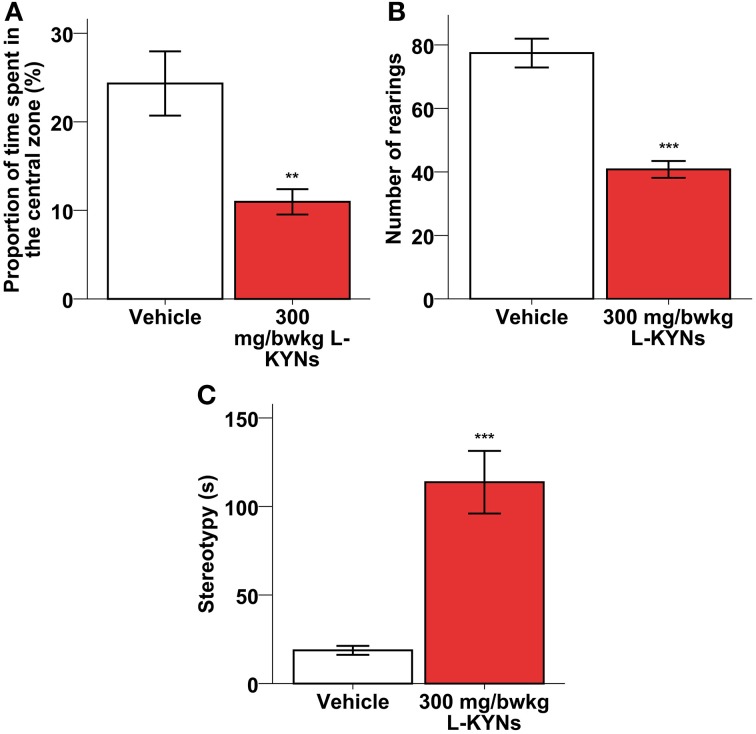
**Effects of L-KYNs treatment on the anxiety-related behaviors of C57Bl/6j mice. (A)** Percentage of time spent in the central area of the OF arena. Treatment significantly diminished the preference for the central zone. **(B)** Number of rearings. Treatment significantly diminished the rearing activity of the animals. **(C)** Time (s) spent expressing stereotypy, e.g., grooming and freezing. Treatment significantly elevated the time of expressing stereotypy behavior. Data are shown as means ± SEM (mANOVA or Mann-Whitney *U*-test ^**^p ≤ 0.01; ^***^p ≤ 0.001; *n* = 19 animals).

A One-Way MANOVA revealed a significant multivariate main effect for treatment, Pillai's Trace = 0.609, *F*_(2, 16)_ = 12.483, *p* = 0.001, partial eta squared = 0.609. The L-KYNs-treated mice spent significantly less time in the highly illuminated central zone of the OF arena (Figure 3A; *p* = 0.006). The number of entries into the central zone does not differ significantly between the two groups (Mann-Whitney *U*-test, *p* = 0.176), however, the time that the animal spend in the central zone, does [independent *T*-test; *t*_(17)_ = 3.160; *p* = 0.006]. These data suggest that the decreased central zone preference is not the result of altered locomotion.

Additionally, the total number of rearings was significantly lowered (Figure 3B; *Z* = −3676, *p* < 0.001), while the time spent in stereotyped grooming and freezing was significantly elevated (Figure 3C; *p* < 0.001).

### Object recognition memory

**Figure 4:**
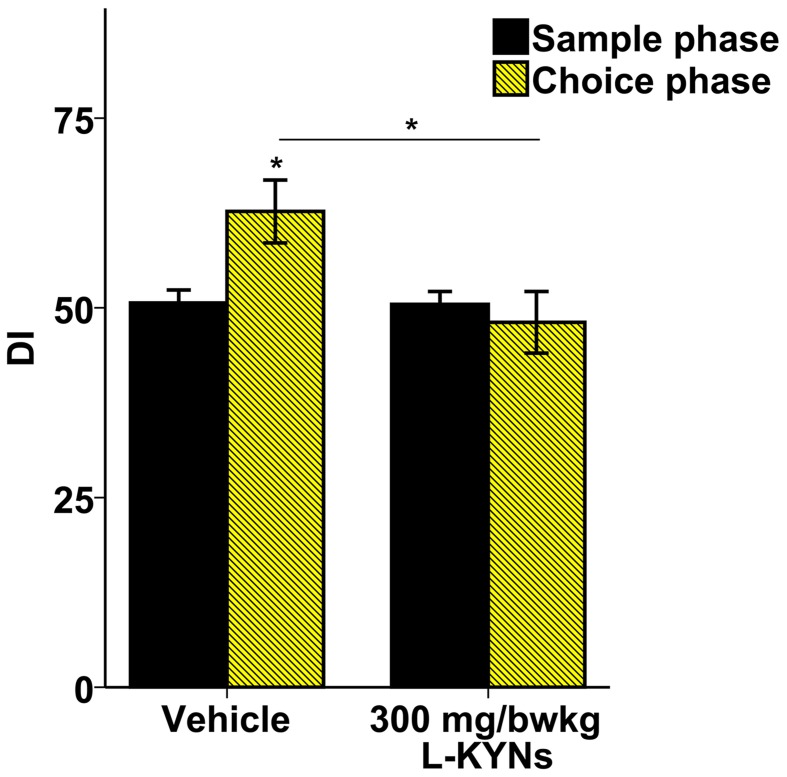
**Effects of L-KYNs treatment on object recognition memory performance, expressed as discrimination index (DI)**. The DI of the control group during the choice phase was significantly elevated as compared with the sample phase. However, there was no such difference in the treated group. The DI of the choice phase for treated group was significantly lower than that for the controls. Data are shown as means ± SEM (paired *t*-test; ^*^p ≤ 0.05; independent *t*-test; ^*^p ≤ 0.05; *n* = 20 animals). [DI: novel × 100/(novel + familiar)].

In the vehicle-treated group during the choice phase mice spent more time exploring the novel object. In contrast, the L-KYNs-treated group spent equal times exploring both objects (Supplementary Figure 2). The treatment altered the OR memory performance expressed as DIs. While the DI of the control group was significantly elevated in the choice phase as compared with the sample phase [*t*_(9)_ = −2.668, *p* = 0.026], there was no statistical differences between the phases in the L-KYNs-treated group. When the choice phases of the two groups were compared, the DI of the treated group proved to be significantly diminished [*t*_(18)_ = −2.325, *p* = 0.032] (Figure 4).

### c-Fos fluorescent immunohistochemistry

**Figure 5:**
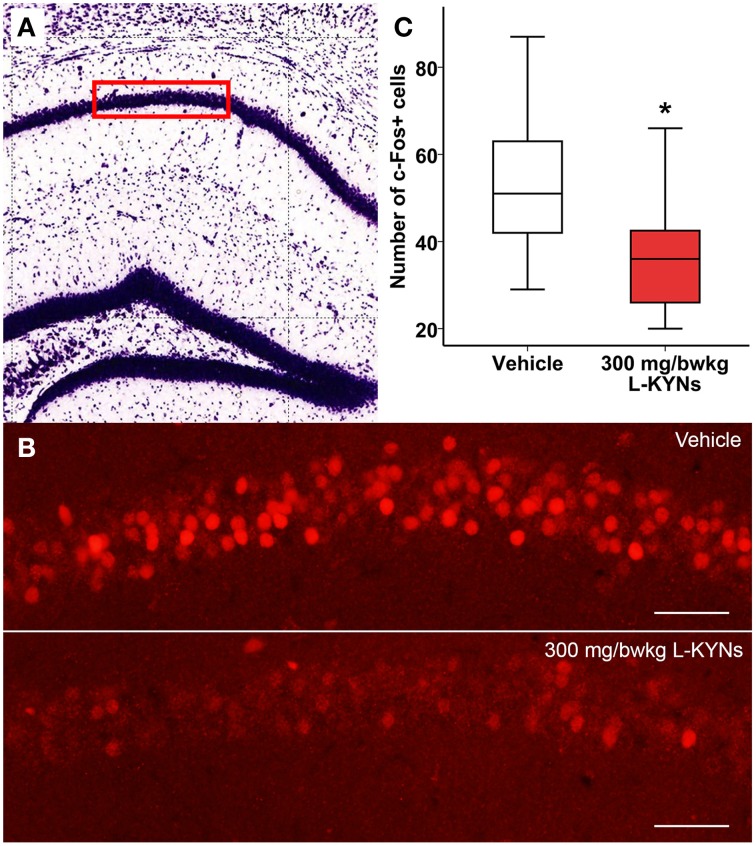
**Effects of L-KYNs treatment on the number of c-Fos^+^ cells in the hippocampal CA1 area of C57Bl/6j mice. (A)** Schematic illustration of the hippocampus. The red box (500 × 140 μm) indicates the captured and analyzed subregion of CA1. **(B)** Representative photomicrographs of c-Fos immunostaining in the CA1. There were a lower number of c-Fos^+^ cells in the L-KYNs-treated group (bottom panel) in comparison with the vehicle-treated group (top panel). Scale bars represent 50 μm. **(C)** Number of c-Fos^+^ cells in the CA1 area. The number of c-Fos^+^ cells was significantly reduced following L-KYNs administration. Data are shown as median, interquartile ranges ± minimum/maximum values (GLMM, ^*^p ≤ 0.05; *n* = 20 animals).

**Figure 6:**
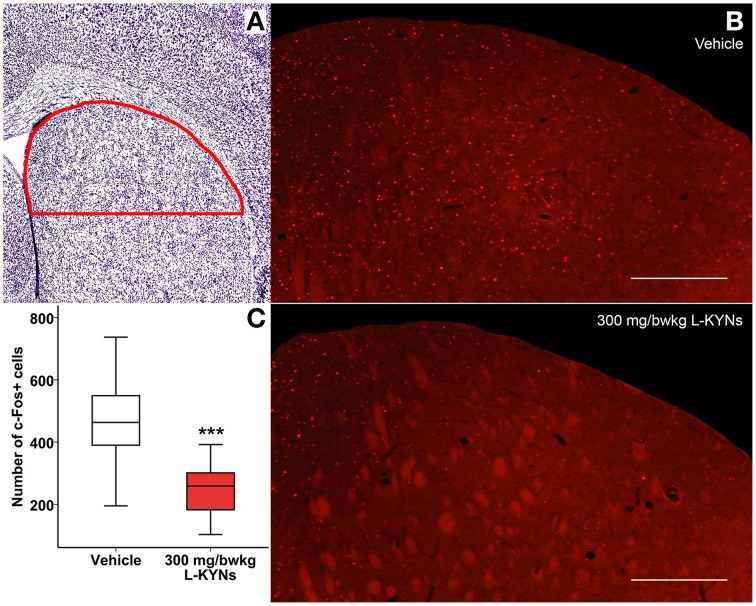
**Effects of L-KYNs treatment on the number of c-Fos^+^ cells in the dorsal striatum of C57Bl/6j mice. (A)** Schematic illustration of the striatum. The red line indicates the captured and analyzed subregion of the dorsal striatum. **(B)** Representative photomicrographs of c-Fos immunostaining in the dorsal striatum. There were a lower number of c-Fos^+^ cells in the L-KYNs-treated group (bottom panel) in comparison with the vehicle-treated group (top panel). Scale bars represent 200 μm. **(C)** Number of c-Fos^+^ cells in the dorsal part of the striatum. The number of c-Fos^+^ cells was significantly reduced following L-KYNs administration. Data are shown as median, interquartile ranges ± minimum/maximum values (GLMM; ^***^p ≤ 0.001; *n* = 20 animals).

In order to study whether L-KYNs can affect the basal c-Fos level in brain structures relevant to the behavioral experiments, we performed c-Fos immunostaining in the CA1 area of the hippocampus and in the dorsal part of the striatum in C57Bl/6 mice. The analyzed subsections of the examined brain areas are illustrated in Figures 5A, 6A. Intensive cytoplasmatic c-Fos immunopositivity was observed in the CA1 pyramidal cell layer, most of the cells expressing the c-Fos protein. There were a lower number of c-Fos+ cells in the pyramidal cell layer of the CA1 subregion in the L-KYNs-treated group in comparison with the vehicle-treated group (Figure 5B). A similar tendency was observed in the dorsal part of the striatum in response to L-KYNs administration. Strong c-Fos immunopositivity was mostly observed in the medial part of the dorsal striatum, whereas cells expressing the c-Fos protein were sporadic in the L-KYNs-treated group (Figure 6B). The differences observed between the vehicle and L-KYNs-treated groups were significant in the hippocampal CA1 area [*F*_(1, 83)_ = 6.501; *p* = 0.013; Figure 5C] and in the dorsal striatum [*F*_(1, 83)_ = 12.701; *p* = 0.001; Figure 6C].

These data suggest that L-KYNs treatment results in a significant reduction in the number of c-Fos^+^ cells in both brain areas relating to our behavioral tests.

## Discussion

This is the first study, which demonstrate that the peripheral administration of L-KYNs does not affect the general ambulatory activity of C57Bl/6j mice, whereas it alters their moving patterns, elevating the movement velocity and resting time. Additionally, it seems to increase anxiety-like behavior, while it completely abolishes the formation of OR memory and also alters the number of c-Fos immunopositive-cells in the dorsal part of the striatum and in the CA1 pyramidal cell layer of the hippocampus.

Because of its poor blood-brain-barrier penetration, systemically administered KYNA cannot directly be used for experiments, examining its role in the CNS. An exogenous administration of L-KYNs can be an efficient way to increase the brain concentration of KYNA. However, systemic administration of L-KYNs may result in the increment of several downstream metabolites (QUIN, 3-HK) of the KP. Furthermore, kynurenine itself is a ligand for aryl hydrocarbon receptors (AHR) through which regulation of immune and inflammatory responses is also possible. We cannot exclude these processes. However, several studies observed increased brain KYNA levels following systemic administration of L-KYNs (Swartz et al., 1990; Chauvel et al., 2012), but increase of other kynurenine metabolites either doesn't occur, or occurs within a certain delay (Speciale et al., 1989; Heyes and Nowak, 1990; Shepard et al., 2003). The main source of QUIN and 3-HK is the activated microglia and the macrophage, infiltrated during inflammatory processes. Furthermore, increased L-KYN influx from the blood exceeds the catabolic capacity of kynurenine 3-hydroxylase in microglia, promoting KYNA production in the astrocytes (Guillemin et al., 2001; Wonodi and Schwarcz, 2010). Guidetti and co-workers proved that in the rat brain KYN is mostly converted into KYNA; only a minor portion is converted into 3-HK and QUIN (Guidetti et al., 1995). The activity of indoleamine-dioxigenase (IDO), the rate-limiting enzyme of the KYN pathway, can be increased by inflammatory signals (Connor et al., 2008). Kynurenine catabolism falls downstream to IDO-mediated processes; inflammatory signals do not influence the effect of exogenous L-KYNs in our experiment.

Based on the literature in our experiments, the short time window (2 h) after L-KYNs administration promotes KYNA production, while the extracellular concentration of the other KP metabolites might be negligible. We may also state that inflammatory signals do not influence our results.

An increased concentration of KYNA in the brain can exert multiple actions on synaptic transmission, resulting in altered behavior. In an OF paradigm, a single systemic injection of L-KYNs (100 mg/bwkg) slightly, but not significantly attenuated the ambulatory activity and significantly decreased the rearing activity of rats (Vécsei and Beal, 1990; Chess et al., 2007).

In our experiments, L-KYNs treatment reduced the rearing activity and altered the moving pattern of the mice. Similar behavioral observations were reported earlier following systemic administration of the non-competitive NMDA receptor antagonist MK-801 to C57Bl/6 mice; the injection of a relatively low dose of MK-801 suppressed the rearing activity and induced abnormal movement velocity (Wu et al., 2005).

Besides the changes in the moving pattern, the L-KYNs treatment significantly attenuated the level of c-Fos expression in the dorsal part of the striatum in C57Bl/6j mice. The sensorimotor cortico-basal ganglia network is responsible for controlling voluntary movements and plays a critical role in determining the movement speed. The fluctuation of dopamine in the dorsal striatum can precisely tune a reference signal, which regulates the movement velocity via control of the input strength of the glutamatergic cortical afferents (Beninger and Olmstead, 2000; Bonsi et al., 2011; Yin, 2014). Experimentally manipulated dopaminergic signaling in rodents leads to an impaired movement velocity control, clearly revealing that the dorsal part of the striatum is critical for the timing of actions (Cousins et al., 1993; Yin, 2014). It has been reported that a nanomolar concentration of KYNA can potently reduce the extracellular level of dopamine in the striatum of unanesthetized rats *in vivo*, via attenuation of the release of glutamate from the glutamatergic cortical afferents by inhibition of the α7nACh receptor (Rassoulpour et al., 2005; Bonsi et al., 2011). For this reason, the attenuated c-Fos expression level in the dorsal part of the striatum could correspond with the abnormalities observed in the moving pattern and movement velocity of the L-KYNs-treated mice.

The OR learning paradigm is a simple and rapid model for detection of the amnestic properties of a neuroactive chemical in rodents (Bertaina-Anglade et al., 2006). In accord with the classical theories of the hippocampal function in rodents, it has been proved that this region is also essential for non-spatial OR memory during the encoding and consolidation phases of the memory process (Hammond et al., 2004; Bertaina-Anglade et al., 2006; Dere et al., 2007; Winters et al., 2008; Assini et al., 2009; Cohen et al., 2013). For this reason, in our experiments the mice were injected 2 h prior to the sample phase of OR task. The peak *de novo* formation of KYNA in the brain of the mice was therefore triggered for the encoding (sample phase) and consolidation (retention interval) phases and not for the retrieval (choice phase) (Swartz et al., 1990; Winters et al., 2008).

In our experiments, the treated mice failed to recognize the novel object in the choice phase. We can assume that the L-KYNs treatment interfered with the formation of recollection of the familiar object (encoding and memory consolidation). Others observed similar results after kynurenergic manipulation. In a radial arm maze paradigm systemic L-KYNs treatment (100 mg/bwkg) impaired the spatial working memory function in rats (Chess et al., 2007). The OR performance can be influenced by a sensory-motoric, attentional or motivational disturbance (Dere et al., 2007). For this reason, the total distance moved as a general ambulatory activity marker was compared. We found no statistical difference in either the sample or the choice phase between the two groups (Data not shown). Concerning the object exploration time, we did not measure decreased exploratory activity in the L-KYNs treated group, in the sample phase. Based on these finding we may conclude that the abolished OR performance is not attributable to altered exploration activity of the animals, instead acquisition and consolidation phases of the memory formation were affected.

Additionally, the treatment significantly attenuated the c-Fos expression level in the pyramidal cells of the CA1 area of the hippocampus. Direct elevation of the KYNA level is known to inhibit the glutamatergic transmission to the CA1 pyramidal neurons predominantly via an α7nACh receptor-dependent mechanism (Banerjee et al., 2012). Others have found that an increased KYNA level can efficiently reduce the excitability of the CA1 stratum radiatum interneurons and lower the GABAergic transmission to the pyramidal cells, via antagonistic actions on α7nACh receptors and NMDA receptors (Alkondon et al., 2011). On top of this, recent findings demonstrated that the administration of specific peptides that disrupt the formation of α7nAch receptor/NMDA receptor coupling complexes in the hippocampus impairs OR memory in mice (Li et al., 2013). For this reason, the attenuated c-Fos expression level in the CA1 area of the hippocampus may parallel the impaired performance of OR in the L-KYNs-treated mice.

L-KYNs treatment increased anxiety-like behavior, significantly decreasing the time spent in the center of the OF, and significantly increasing stereotypy. Similar observations have been reported in rats, where systemic L-KYNs treatment induced an increased level of anxiety in an elevated plus-maze test (Vécsei and Beal, 1990). Additionally, the direct i.c.v. administration of KYNA resulted in ataxia and stereotypy in a dose-dependent manner (Vécsei and Beal, 1991). Chronic administration of L-KYNs causes long-term disturbances in rodent behavior. Following pre- and post-natal exposure to L-KYNs, adult rats exhibited an impaired performance in a behavioral test linked to the hippocampal function (Pocivavsek et al., 2012), while adult mice demonstrated an enhanced sensitivity to D-amphetamine-induced increase in locomotion activity following neonatal L-KYNs injections (Liu et al., 2014). Moreover, chronic elevation of brain KYNA level during development in rats, caused cognitive and behavioral disturbances in the adult animals (DeAngeli et al., 2015).

Under our experimental conditions, a single exposure to L-KYNs led to behavioral disturbances and a reduction in the level of expression of a transcriptional factor c-Fos in different subcortical areas. The c-Fos protein is an immediate early gene product applied as an almost universal neuronal activity marker. In the nucleus, c-Fos can mediate long-term responses due to enhanced neurotransmission, including the expression of tissue-specific genes or information storage (Kaczmarek, 2002). The transcription of *c-fos* is controlled through an increase in the intracellular concentration of Ca^2+^. During plastic processes in the murine brain, the activation of Ca^2+^-permeable NMDA receptors and AMPA receptors is the most determinant, and the influence of Ca^2+^-permeable α7nACh receptors can also be considerable (Sagar et al., 1988; Séguéla et al., 1993). The activation of NMDA or non-NMDA glutamate receptors was found to induce a rapid and dramatic Ca^2+^-dependent increase in *c-fos* mRNA in the dentate gyrus neurons, *in vitro* (Lerea et al., 1992). Moreover, the activation of nACh receptors induces the rapid transcription of *c-fos* mRNA in non-dividing, neuronally differentiated PC12 cells in a Ca^2+^-dependent manner (Greenberg et al., 1986). Beyond its transcriptional role, c-Fos protein is a generally accepted neuronal activity marker. c-Fos expression level sensitively correlates with neuronal activity after physiological stimuli or in pathological states (Kaczmarek, 2002). Kynurenergic manipulation proved to attenuate the pathological elevation of c-Fos protein expression in different experimental models (Knyihar-Csillik et al., 2008). The direct intraplantar administration of KYNA or the systemic administration of its precursor L-KYNs, can effectively reduce the chemically-induced c-Fos expression level in various pain models (Zhang et al., 2003; Knyihár-Csillik et al., 2007a,b).

The multiple action of an elevated brain KYNA level on neurotransmission may converge to an altered c-Fos level and concomitant changes in neural function and behavior. Fluctuations in the level of expression of c-Fos in the brain are therefore to be expected following to L-KYNs administration. It might also be suggested that a single treatment affects the mouse brain plasticity owing to transcriptional changes.

## Conclusions

We have demonstrated the influence of treatment with a single high dose of L-KYNs on the behavioral and neuronal activity in C57Bl/6j mice *in vivo*. The main results from the present experiments indicate that L-KYNs treatment does not affect the general ambulatory activity, but alters the moving pattern of the mice, elevating the moving velocity, and increasing the proportion of resting time and anxiety-like behavior. Furthermore, the treatment abolishes the formation of OR memory. These behavioral abnormalities may be related to the altered basal c-Fos protein expression and the imbalance of the striatal and hippocampal neuronal activity.

The methods used during our experiments may be valuable tools for future studies of the pathway of the KYN metabolism.

### Conflict of interest statement

The authors declare that the research was conducted in the absence of any commercial or financial relationships that could be construed as a potential conflict of interest.

## Supplementary material

Supplementary Figure 1 **Unique objects in the OR task were constructed from Lego blocks that differed in shape and color**.

Supplementary Figure 2 **Object exploration time in the sample and choice phases in both animal groups**.
