# Supplementary figures and images for: Systemic L-Kynurenine sulfate administration disrupts object recognition memory, alters open field behavior and decreases c-Fos immunopositivity in C57Bl/6 mice
Source: Front Behav Neurosci. 2015 Jun 16;9:157. doi: 10.3389/fnbeh.2015.00157 (PMC4468612; doi:10.3389/fnbeh.2015.00157)

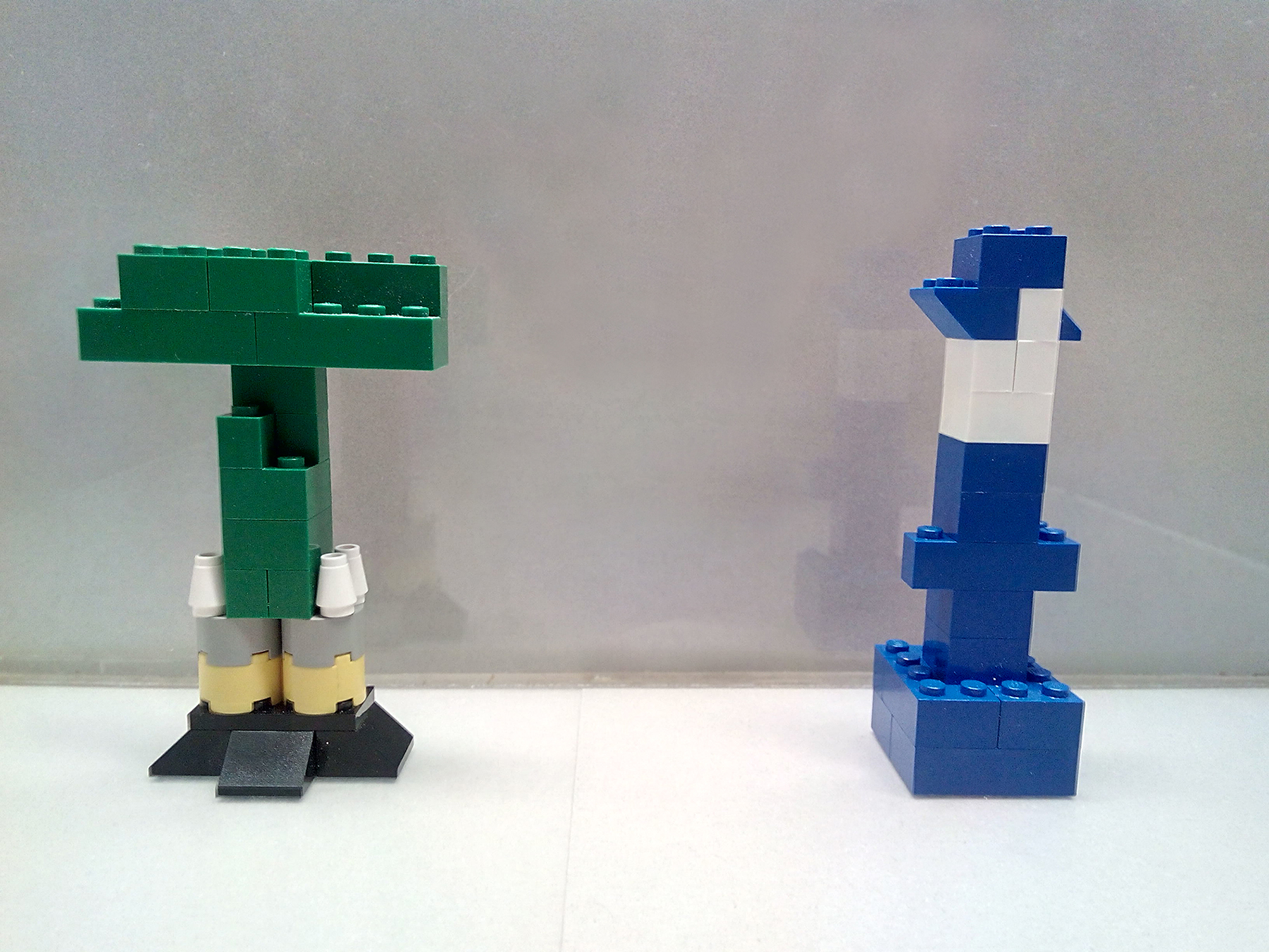

Supplement: Supplementary Figure 1 — Unique objects in the OR task were constructed from Lego blocks that differed in shape and color. [file Image1.TIF]

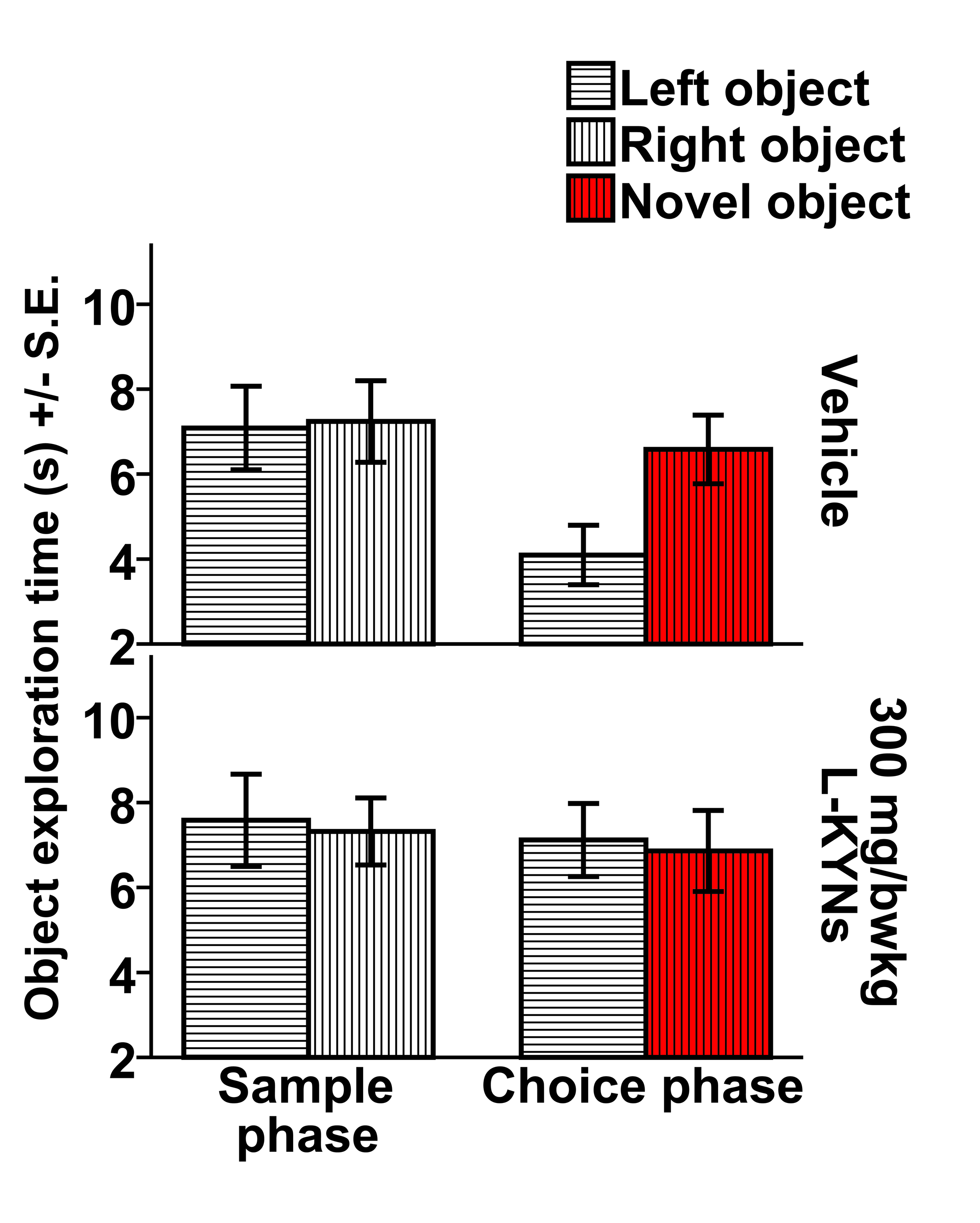

Supplement: Supplementary Figure 2 — Object exploration time in the sample and choice phases in both animal groups. [file Image2.TIF]
